# Individual differences in dual-target RSVP task performance relate to entrainment but not to individual alpha frequency

**DOI:** 10.1371/journal.pone.0178934

**Published:** 2017-06-12

**Authors:** Cornelia Kranczioch

**Affiliations:** 1 School of Medicine and Health Sciences, Department of Psychology, Neuropsychology Lab, University of Oldenburg, Oldenburg, Germany; 2 Research Center Neurosensory Science, University of Oldenburg, Oldenburg, Germany; University of Groningen, NETHERLANDS

## Abstract

The attentional blink (AB) paradigm is widely used to study visual temporal attention. An important feature of the standard AB paradigm is repetitive visual stimulation, more precisely the rapid serial visual presentation (RSVP) of numerous distracters interspersed with two targets. The RSVP stream is likely to result in entrainment of visual cortex, which has been suggested to negatively affect target identification in the AB paradigm. The present EEG study tested this idea with an inter-individual differences approach. AB task performance and measures of entrainment were derived from 51 participants. Other than predicted, moderate positive correlations were observed for inter-trial coherence and performance, but only for targets not immediately preceded by other targets. A positive correlation with power was evident for targets presented in the critical AB time window. In a second step, it was tested whether the distance between individual alpha frequency and RSVP frequency mediated correlations with inter-trial coherence, as entrainment of the visual cortex through repetitive visual stimulation is particularly effective when the frequency of the stimulation matches the individual alpha frequency. However, no evidence was found supporting such link. While compatible with a number of findings related to the AB and to visual entrainment, the findings of the present study do not provide evidence for the notion that entrainment to the RSVP stream creates a neural environment unfavourable for detecting targets an RSVP stream.

## Introduction

In many situations are we faced with quickly incoming information, an obvious example is driving a car. That we are likely to miss a considerable amount of this information becomes evident in the more controlled situation in which two visual target stimuli are presented in close succession and in the context of a rapid sequence of visual distracters, and where the second target is frequently missed when presented within 200 to 500 ms of the first target [1,2]. This phenomenon has been termed the attentional blink (AB) [3]. Even though the rapid serial visual presentation (RSVP) distracter stream is not essential for observing the AB, the importance of this stream of non-targets is evident in the finding that it amplifies the AB [4–6]. Another interesting characteristic of the AB is that it varies across individuals, with less than 10% of participants displaying no or only a very small AB even in the presence of a distracter stream [7,8].

Repetitive visual stimulation results in brain responses in the frequency of the stimulation, the so-called steady-state visual evoked potential (ssVEP) [9,10]. This frequency locking or entrainment has been shown to be particularly prominent when the stimulation frequency is close to the frequency at which on average alpha activity at rest is highest [11], or, even more fine scaled, when the stimulation frequency is matched to the individual alpha peak, also known as individual alpha frequency or IAF [12,13]. The presentation rate of the RSVP distracter stream in a typical AB experiment is ten items per second or 10 Hz and thus falls in the centre of resting alpha activity (7 to 13 Hz). For some participants this results in a rhythmic stimulation very close to their IAF while for others RSVP frequency and IAF will diverge.

With regard to the AB paradigm, Hanslmayr and colleagues [14] postulated that the entrainment of the visual system to the RSVP stream creates a neural environment unfavourable for detecting the second target in the RSVP stream. This idea is supported by the finding that both T1 and T2 identification in the AB task can be improved when entrainment to the RSVP stream is reduced by introducing temporal jitter to the stream ([15], but see [16]). Hanslmayr et al. [14] furthermore grant that because no study has yet examined how first target processing affects the components of the postulated unfavourable neural environment or internally oriented brain state, they cannot rule out the possibility that it is amplified by T1 processing.

When bringing together the basic idea of the unfavourable role of entrainment to the RSVP stream [14] in the AB task and the findings regarding entrainment and IAF [11–13] one can derive two predictions: First, in a typical AB setting, individuals will display differences in the degree of entrainment to the RSVP stream that correlates with inter-individual differences in AB task performance. Stronger entrainment should be associated with poorer performance. Second, the relationship between AB task performance and entrainment is mediated by the distance between IAF and RSVP frequency (ΔF). Partialling out the variance accounted for by ΔF from the correlations between measures of entrainment and AB task performance should significantly reduce these correlations. The present study tested these predictions.

## Materials and methods

The institutional review board (IRB) *Kommission für Forschungsfolgenabschätzung und Ethik der Carl von Ossietzky Universität Oldenburg* approved this research in its meeting on February 10 2016 (approval number Drs. 5/2016).

### Participants

Participants were students of Oldenburg University, recruited through notifications at the university’s virtual black board. A total of 57 participants took part in the study. Six participants were excluded from further analysis because of they showed either no clear or two alpha peaks. The final sample of 51 participants consisted of 25 men and 26 women with a mean age of 23.8 years (range = 20–34 years). Of this sample, based on the Edinburgh Handedness Inventory four were identified as ambidextrous and four as left. All participants were free of neurological or psychiatric illnesses. Before the start of the experiment, all participants gave written informed consent according to the Declaration of Helsinki. Participants received a monetary compensation of 8 EUR/hour.

### Stimuli and setup

Participants performed a classical attentional blink (AB) task. The experiment was run using Presentation software version 18.2 (Neurobehavioral Systems, Inc. Albany, CA). Targets were digits (1–9), distracters were capital letters (all letters of the alphabet except I, J, O, Q). Stimuli were presented in Luicida Sans Unicode font at a rate of 10 per second. Stimulus colour was white and background colour was dark grey. Stimulus size was 8 cm (height) by 4.4 to 8 cm (width) or 2.6 (height) by 1.4 to 2.6 (width) degrees of visual angle. Each RSVP trial consisted of 31 stimuli with stimuli drawn randomly with replacement with the restriction that no two subsequent stimuli would be identical. Stimuli were presented for 66.67 ms with an ISI of 33.33 ms, refresh rate of the monitor was 60 Hz.

**Fig 1:**
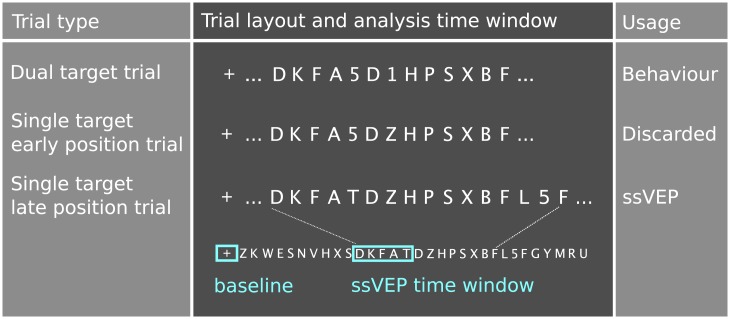
Schematic depiction of the trial layout for the three different trial types. Targets were digits, distracters were letters. A trial could contain either one or two targets. If it contained only one target the target appeared either in an early position or a late position in the RSVP stream. Trials with a single target in a late position (bottom) were used to extract the ssVEP. For statistical analysis data were extracted from the time range corresponding to the presentation of the 11^th^ to 15^th^ distracter in the RSVP stream. The baseline interval was extracted from the pre-RSVP stream fixation period.

The experiment was split in six blocks with 30 sec breaks in-between. Blocks consisted of 15 trials containing two targets (T1 and T2, five trials per T2 lag), five trials with a single target in an early stream position and ten trials with a single target in a late stream position (see Fig 1). Behavioural performance was derived from dual target trials. RSVP-related analysis of EEG data was based on single target trials with late target. Trials with single early targets were included in the design to avoid any potential confounds arising from clustering single targets at the end of the RSVP stream in single target trials. This trial type was not included in the analysis. The first target in dual target trials and the early single target were presented at stream positions 14 to 17. The single late target was presented at stream positions 24–31. In dual target trials, T2 was presented at either lag 1, 2, or 7, corresponding to the +1, +2, or +7 position relative to T1. After the offset of the stream participants were prompted to enter up to two target digits they had identified with a keyboard. Responses were considered correct irrespective of order. After the participant confirmed her answer(s) by pressing the Enter key, a new trial started with a fixation cross of 900 to 1100 ms duration (randomized across trials, 50 ms steps). The AB experiment started with a practice block of six dual target trials and six single target trials. Before and after the attentional blink experiment resting EEG was recorded with respectively 2 minutes eyes open and two minutes eyes closed.

### EEG data collection and preprocessing

Participants were seated comfortably in a dimly lit, sound attenuated booth. The monitor used for stimulus presentation was mounted outside the booth at a distance of about 175 cm from the participant’s eyes. Electroencephalographic (EEG) signals were continuously recorded from 64 Ag/AgCl electrodes mounted on an elastic cap (EASYCAP GmbH, Herrsching, Germany) using a BrainAmp amplifier (BrainAmp, Brain Products GmbH, Gilching, Germany). Electrodes were positioned according to a customized equidistant layout covering the scalp. A central, frontopolar channel served as ground. All channels were recorded against a nose-tip reference. Data were sampled at 500 Hz and recorded with a 0.016 Hz highpass and a 250 Hz lowpass filter. Electrode impedances were kept below 20 kΩ.

Data were analyzed offline using MATLAB R2012a (The Math-Works, Inc., Natick, Massachusetts, USA) and EEGLAB 12.0.2.4b software (http://sccn.ucsd.edu/eeglab/, [17]). Independent component analysis (ICA) was used for artifact correction [18]. In a first step, original data were filtered using a 1 Hz highpass filter (windowed sinc FIR-filter, Hamming windowed, filter order 16500) and a 40 Hz lowpass filter (windowed sinc FIR-filter, Hamming windowed, filter order 826). Data were segmented into 2-second epochs. Atypical and rare artifacts were automatically identified using routines from EEGLAB. The result was confirmed by visual inspection and contaminated epochs were rejected before running ICA. In a second step, ICA decomposition results were imported to the original, continuous data sets filtered from 0.1 Hz (0.5 Hz for resting data, windowed sinc FIR-filter, Hamming windowed, filter order 15500/13750) to 30 Hz (windowed sinc FIR-filter, Hamming windowed, filter order 826), and independent components (ICs) representing prototypical artifacts such as eye movements or ECG were identified and removed [19]. These steps were run identically for the concatenated resting EEG sessions and for the AB experiment.

**Fig 2:**
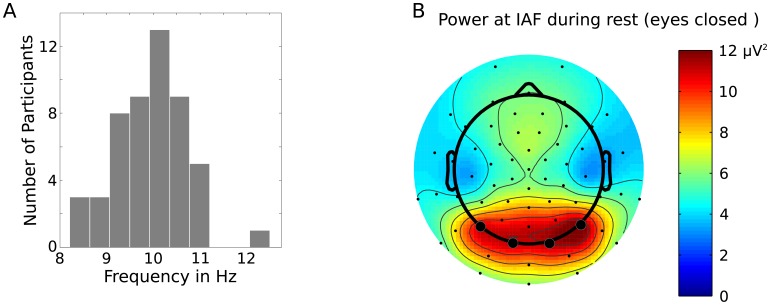
IAF histogram and topography. (A) Histogram of all IAF values for the first resting session. (B) Topographical distribution of resting power at the IAF across the group for the first resting session. Black circles indicate the position of the electrodes selected for statistical analysis.

For extracting the IAF the ICA pruned resting data were split into four segments corresponding to the two eyes open and the two eyes closed periods of resting EEG. Segments were screened for residual non-stereotyped artefacts using the EEGLAB function pop_rejcont and segments containing artefacts were rejected. In five participants, a single bad channel was identified, these channels were interpolated using spherical spline interpolation. FFT was run on 2-second segments without overlap (EEGLAB function spectopo). Data were five times oversampled to achieve a frequency resolution of 0.39 Hz. The IAF was extracted from the eyes closed data from both resting EEG sessions as the frequency with maximal amplitude at any channel in the frequency range from 7.03 to 13.28 Hz. Resting power was calculated as the mean power of four occipito-parietal channels forming the region of interest (ROI, cf. Fig 2).

For analysis of the ssVEP, ICA cleaned data were segmented into 2500 ms epochs, covering a 500 ms baseline and the first 2000 ms of the RSVP distracter stream in late single target trials. Segments were baseline corrected. Epochs containing residual atypical and rare artifacts were again automatically identified using routines from EEGLAB and were rejected. An FFT-based time-frequency analysis was then performed using the EEGLAB newtimef function to extract 10 Hz total power and inter-trial coherence (ITC) for the whole epoch as measures of entrainment over time. The parameters of the function were set such that power data were dB scaled and baseline was corrected with a baseline ranging from -200 to 0 ms (for more details on the baseline settings used see S2 File). To derive ERP or evoked power the analysis was repeated but the time frequency analysis was based on the individual ERP. That is, the single trial average was used instead of single trials. For statistical analysis, mean power values and mean ITC were extracted from the same four occipito-parietal ROI channels used for resting power for the time range 900 ms to 1900 ms post RSVP onset. The time range was set to exclude any RSVP onset.

### Statistical analysis

The presence of lag-dependent modulations of T2|T1 identification rates were analyzed in a repeated measures ANOVA with factor T2 lag (lag 1, lag, 2, lag 7). Test-retest reliability of IAF values and all other correlations were calculated with Pearson correlations. To account for type-one error inflation in case of multiple correlations a Bootstrap statistic with 1000 samples as implemented in SPSS 24 was run to calculate 95% confidence intervals. A correlation was only considered significant if the respective confidence interval did not include 0. Partial correlations were used to test for a mediating effect of ΔF, with ΔF defined as *ΔF = abs(IAF-10)*. All data points behind means and correlations can be found in supplementary materials S1 File.

## Results

### Behaviour

T1 identification rate in dual target trials was 86.6% (SE = 1.1). In single target trials T1 identification rate was 95% (SE = 0.6) when T1 was presented early in the stream and 96.4% (SE = 0.5) when T1 was presented late in the stream. T2|T1 identification rates displayed a classical AB pattern. Mean identification rates were 83.9% (SE = 2.3) for T2 lag 1, 44.2% (SE = 3.5) for T2 lag 2, and 85.6% (SE = 1.8) for T2 lag 7. The one-way ANOVA analysing lag-depended modulations in T2|T1 performance yielded a significant main effect of lag, F(1,50) = 107.0, p < .0001, partial eta squared = .68. Post-hoc T-tests indicated significant differences between T2 lag 1 and T2 lag 2 (t(50) = 13.1, p < .0001) and T2 lag 2 and T2 lag 7 (t(50) = -11.4, p < .0001), but not between T2 lag 1 and T2 lag 7 (t(50) = -.59, p = .56).

### Alpha activity at rest

Mean IAF was 10.07 Hz (SD = 0.88) for the first resting EEG session and 9.89 Hz (SD = 0.73) for the second resting session. Test-retest reliability was highly significant with r = .88 and p < .0001, indicating that IAF could be reliably measured. Because of the high test-retest reliability, for all subsequent analyses IAF data from the first and second session were averaged. Fig 2A shows a histogram of the average IAF values. Fig 2B depicts the corresponding topographical distribution of average resting power at the IAF across the group. As would be expected, IAF and power at IAF extracted from the ROI were negatively correlated in that higher IAF went along with lower power (r = -.40, p = .004).

### Entrainment during RSVP distracter stream processing

**Fig 3:**
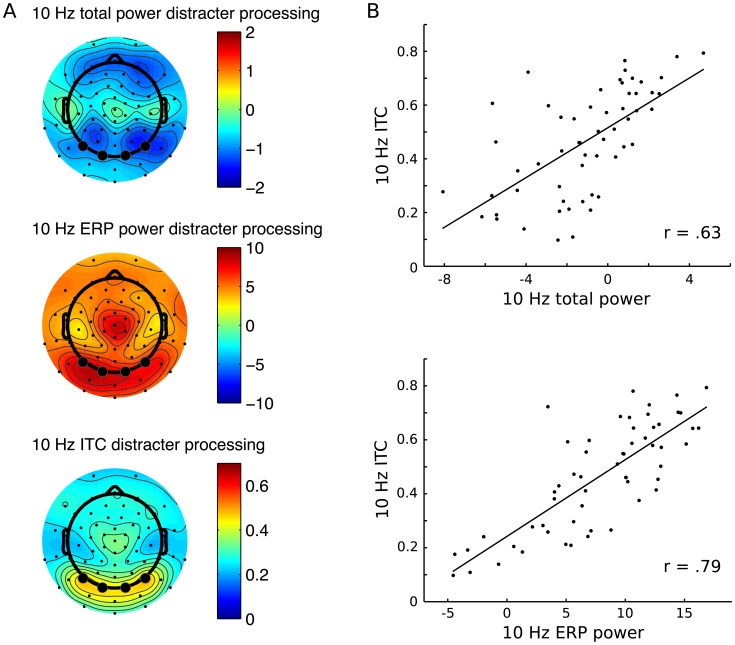
Power and ITC at 10 Hz as measures of entrainment. (A) Top: Total power at 10 Hz during the RSVP stream relative to the pre-RSVP baseline. The plot depicts the mean total power from 900 to 1900 ms post RSVP stream onset. Middle: Mean ERP power at 10 Hz from 900 to 1900 ms post RSVP stream onset relative to pre-RSVP baseline. Bottom: ITC at 10 Hz, mean value from 900 to 1900 ms post RSVP stream onset. (B) Scatter plots and regression lines of 10 Hz ITC and 10 Hz total power (top) and 10 Hz ITC and 10 Hz ERP power (bottom).

At the group level, during RSVP distracter stream processing, total power at 10 Hz was reduced relative to the pre-task baseline (Fig 3A top). 10 Hz ERP power and ITC were enhanced at parieto-occcipital scalp regions as compared to other scalp areas (Fig 3A middle and bottom). On the individual level, it was evident that in spite of the negative group mean for 10 Hz total power, values ranged from about– 8 dB to about 4 dB. As depicted in Fig 3B (top), higher 10 Hz total power in the ROI was associated with higher 10 Hz ITC (r = .63, p < .0001). The same applied for 10 Hz ITC and 10 Hz ERP power, r = .79, p < .0001 (Fig 3B, bottom). Neither 10 Hz total power, 10 Hz ERP power, nor 10 Hz ITC correlated significantly with ΔF (respectively r = .01, p = .93; r = .12, p = .39; r = .10, p = .51).

### Correlation between RSVP task performance and measures of entrainment

**Fig 4:**
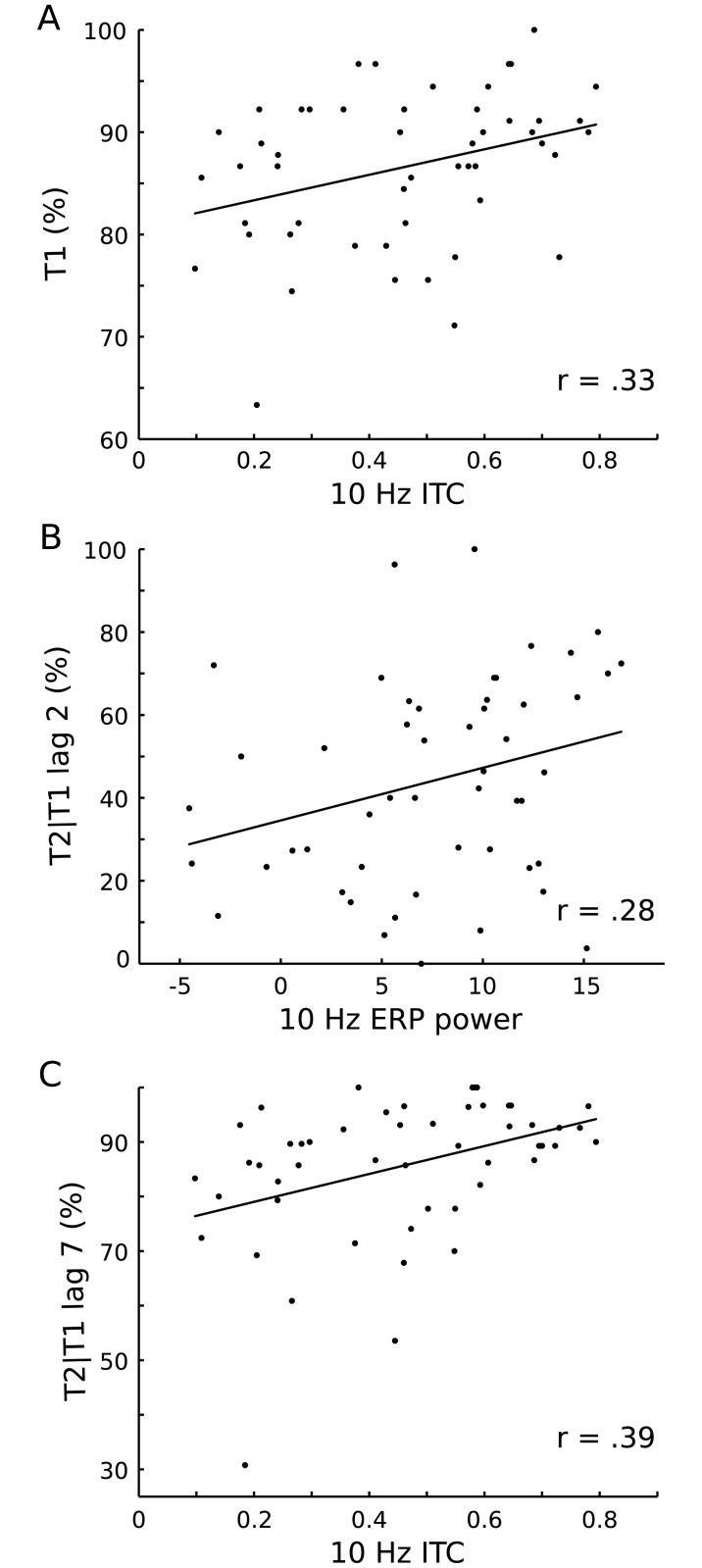
Significant correlations between 10 Hz ITC and task performance. (A) T1 identification rate as a function of 10 Hz ITC. The black line corresponds to the linear regression line. (B) Identification rate for T2|T1 lag 2 as a function of 10 Hz ERP power. The black line corresponds to the linear regression line.

ROI 10 Hz power and ITC values were correlated (two-tailed) with identification rates for T1, T2|T1 lag 1, T2|T1 lag 2, and T2|T1 lag 7. Results are summarized in Table 1. To account for type-one error inflation Bootstrap statistics as implemented in SPSS 24.0 (1000 samples), were run for each correlation. Significant moderate positive correlations were observed for ITC and T1 identification rate, for ITC and T2|T1 lag 7 identification rate, and for T2|T1 lag 2 identification rate and ERP power. Trends (all p < 0.062) in the same direction were evident for ERP power and for T1 and T2|T1 lag 7 identification rates and for total power and for T1 identification rates. To summarize, in contrast to our prediction, stronger entrainment as reflected in larger ITC values and ERP power was related to better performance (cf. Fig 4).

**Table 1:** Correlation of 10 Hz total power, ERP power and ITC.

	T1	T2|T1 lag 1	T2|T1 lag 2	T2|T1 lag 7
10 Hz total power	r = .22(*)	r = .09	r = .22	r = .23
	p = .062	p = .55	p = .12	p = .11
10 Hz ERP power	r = .27(*)	r = .23	r = .28*	r = .27(*)
	p = .057	p = .12	**p = .04**	p = .056
10 Hz ITC	r = .33*	r = .13	r = .18	r = .39**
	**p = .018**	p = .36	p = .21	**p = .005**

** p < 0.01,

* p < 0.05,

^(^*^)^ p<0.10

The scatter plots presented in Fig 4 indicate the presence of respectively one potential outlier in T1 and in T2|T1 lag 7 identification rates. Removing these outliers reduced the corresponding significant correlations but did not eliminate them (result in S1 Table). Moreover, on general, ITC values are influenced by the number of trials entering the calculation, with ITC being the larger the less trials [20]. An additional analysis was conducted to explore whether ITC and trial number were negatively correlated in the present data set, where number of trials ranged between 52 and 60 (mean 56.1, SD = 1.9). In contrast to the expectation, a moderate positive correlation (r = .28, p = .48) was observed, that is, more trials were associated with larger ITC (scatter plot in S1 Fig). To rule out that this positive correlation might drive the significant correlations observed for ITC and target performance the two correlation analyses were repeated with trial number partialled out. The resulting partial correlation coefficients were only slightly smaller than the original correlation coefficients (results in S1 Table), indicating that number of trials is indeed no relevant factor here.

Correlations of target performance and 10 Hz power and ITC indicated a positive correlation between 10 Hz ERP power and T2|T1 lag 2 identification rate. To test whether a correlation was also evident for the AB, AB magnitudes were calculated. Different approaches for calculating AB magnitude have been suggested. One popular measure is the difference between T2|T1 lag 7 and T2|T1 lag 2 identification rates [21], another the difference between T1 identification rate at lag 2 and T2|T1 lag 2 identification rate, divided by T1 identification rate at lag 2 [7]. Significant correlations were observed for neither measure (Table 2).

**Table 2:** Correlation of 10 Hz total power, ERP power and ITC with T2|T1 lag 2 identification rate and AB magnitudes.

	T2|T1 lag 2	AB Magnitude [21]	AB Magnitude [7]
10 Hz total power	r = .22, p = .12	r = -.10, p = .50	r = -.18, p = .21
10 Hz ERP power	r = .28*, **p = .04**	r = -.14, p = .34	r = -.25, p = .08
10 Hz ITC	r = .18, p = .21	r = .03, p = .86	r = -.13, p = .36

* p < 0.05,

### Influence of ΔF

Partial correlations were used to test whether the correlations observed between ITC and T1 lag 1 performance and ITC and T2|T1 lag 7 performance and between ERP power and T2|T1 lag 2 performance were mediated by ΔF. Controlling for the effect of ΔF did not reduce the correlations (r_ITC,T1lag1/ΔF_ = .35, p = .013; r_ITC,T2|T1lag7/ΔF_ = .39, p = .005). Subsequent exploratory analyses confirmed that also the direct correlations between performance and ΔF were not significant (all r(abs) < .16, all p > .27).

## Discussion

This study applied an inter-individual differences approach to test the predictions that entrainment to the RSVP distracter stream relates negatively to target identification in the AB task and that the relationship between entrainment and AB task performance is mediated by ΔF, the distance between the RSVP frequency and the IAF. While significant moderate correlations were observed, the direction of the correlations was opposite to the predicted direction. Moreover, correlations with ITC were restricted to T1 and to T2 lag 7 identification rates. An additional positive correlation was observed for ERP power and T2 lag 2 identification rate. Other than predicted, correlations were not mediated by ΔF.

The results of the present study do not provide evidence that at the inter-individual level entrainment to the 10 Hz distracter stream typical for the AB paradigm or larger power during the distracter stream period are negatively related to AB task performance. On the contrary, the results indicate that performance in the AB paradigm benefits from entrainment. Though in contrast to a number of AB-specific findings and theories [7,14,15], the observation of a positive correlation between entrainment and performance is in line with findings from detection tasks. Here it has been reported that a visual target presented in phase with an entraining sequence—as is the case for targets in the AB paradigm—is more easily detected than a target presented with a phase shift, with the difference being the more pronounced the longer the entraining sequence [22]. The positive effect of a longer period of entrainment for identification and processing of a single target has also been demonstrated in the RSVP-based Attentional Awakening paradigm [23–25]. But also several findings from AB studies are difficult to integrate with the idea that entrainment or power increases to the RSVP stream are detrimental to AB task performance: For instance, taking an intra-individual differences approach Janson and colleagues [4] observed an interaction between correct report of T2 and total power at the RSVP and the IAF frequencies: while high power in the RSVP frequency in the pre-target distracter stream was linked to correct report of T2, the opposite pattern emerged for IAF power. As another example, the positive effect of introducing temporal jitter in the RSVP stream on AB task performance [15] could not be replicated in a later study [16].

Importantly, in the present study a significant link between 10 Hz entrainment and performance was only observed for the T1 position and for the T2 lag 7 position. This suggests that while entrainment to the distracter stream is a relevant factor for target identification in the AB paradigm, its relative importance depends on the target position: While significantly contributing to identification success of targets preceded by longer uninterrupted periods of task irrelevant items, its importance is reduced when the RSVP stream and thus entrainment to the stream are interrupted by target processing [26]. It can be speculated that this interruption leaves additional room for other factors—both intra-and inter-individual—to gain importance. This assumption of a differentiation is supported by the finding that T2|T1 lag T2 performance correlated with ERP power in the absence of a parallel significant correlation or even a trend for ITC. Such additional factors could for instance be executive working memory [27,28] or attentional investment to target identification (e.g. [29,30]) or most likely, given the considerable number of factors found to influence the AB, a combination of several factors (for a recent review of inter-individual factors see [31], for more general reviews on the AB see for instance [21,32]). The assumption that the interruption of the task irrelevant distracter stream by a target leaves room for other factors than entrainment to the stream to gain importance and thus to determine the fate of a subsequent target is also compatible with the suggestion that it might be in particular T1 processing and associated detrimental changes in the current brain state that are crucial for the AB phenomenon [14]. It is not in line though with the suggestion of a critical role of pre-target entrainment in the alpha range through the RSVP stream for the full manifestation of these changes [14]. Interestingly, a recent study by Shapiro et al. [33] comes to the opposite conclusion. Here, in two experiments the frequency of the RSVP distracter stream was manipulated while keeping the distance between T1 and T2 as constant as possible. RSVP frequency was set to 6.26 Hz (theta), 10.3 (alpha), 16.0 (beta) and 36.0 Hz (gamma), with the prediction that the AB should be particularly pronounced for an RSVP stream in the alpha-beta frequency range. The results of the experiments confirmed this prediction and were interpreted as showing that the AB is critically tied to the alpha-beta frequency range of the entraining RSVP. However, as this was a purely behavioural study it is impossible to say whether the different entraining frequencies resulted in a difference in the proposed detrimental changes in the post T1 brain state. Moreover, it is not clear whether the special role of alpha-beta frequency range entrainment implied by the results of Shapiro et al. [33] persists when the content of the entraining distracters can be easily ignored at all frequencies as in the case of a single repeatedly presented distracter [4] or when the level of difficulty is adjusted across RSVP frequencies not only for target processing but also for distracter processing.

The observed link between entrainment to the RSVP stream and AB task performance was not mediated by ΔF, nor did ΔF directly correlate with AB performance. The latter is in line with the finding reported for a considerable smaller group of participants [16] that AB magnitude does not differ between participants with IAF around 10 Hz (9–10.7 Hz) and IAF deviant from 10 Hz (lower than 9, higher than 10.7, with a range of 8.8–11.7 Hz for both groups). The results of the present study indicate that this earlier null-finding cannot be explained by the small sample size or by the creation of somewhat arbitrary groups.

It remains an open question why we did not observe a negative correlation between 10 Hz power and ITC with ΔF, as would be expected based on the ssVEP literature focusing on stimulation frequencies in the alpha range [11–13]. One important reason could be a fundamental difference between the pure ssVEP studies and the present one: while former had their participants passively view flickering light, latter required active processing of meaningful elements making up an RSVP stream. This active processing might blur or interfere with the rules of entrainment derived from purely passive situations. Future studies should aim to combine purely passive and active settings within one study and with the identical sample of participants to shed light on this issue.

To summarize, the findings of the present study suggest a positive link between entrainment to the RSVP stream and ERP power during the RSVP stream, and performance in the AB paradigm. The entrainment link appears restricted to targets not immediately preceded by other targets and suggests that other than predicted stronger entrainment to the RSVP stream relates here to better performance. In combination with findings from other studies the findings of the present study underline the necessity to further refine the notion that entrainment to the RSVP stream creates a neural environment unfavourable for detecting targets in the RSVP stream.

## Supporting information

S1 Fig Scatter plot and regression line for ITC. Scatter plot and regression line for ITC at 10 Hz and number of trials for the present data set. The correlation is significant with r = .29, p = 0. 048.
(DOCX)

S1 File Data points behind means and correlations. The Excel file contains the data points behind the reported means and correlations.
(XLSX)

S2 File Settings for optional baseline parameters used in EEGLAB function newtimef. Listed are the settings for optional baseline parameters as used for data analysis in the present study.
(DOCX)

S1 Table Correlation coefficients and *p*-values. Correlation coefficients and *p*-values for 10 Hz ITC with T1 identification rate and T2|T1 lag 7 identification rate.
(DOCX)
