# Supplementary material for: Individual differences in dual-target RSVP task performance relate to entrainment but not to individual alpha frequency
Source: PLoS One. 2017 Jun 12;12(6):e0178934. doi: 10.1371/journal.pone.0178934 (PMC5467839; doi:10.1371/journal.pone.0178934)
Supplement: S1 Fig — Scatter plot and regression line for ITC at 10 Hz and number of trials for the present data set. The correlation is significant with r = .29, p = 0. 048. (DOCX) [file pone.0178934.s001.docx]

**S1 Fig. Scatter plot and regression line for ITC.** Scatter plot and regression line for ITC at 10 Hz and number of trials for the present data set. The correlation is significant with r=.29, p=0. 048.
