# Supplementary material for: Individual differences in dual-target RSVP task performance relate to entrainment but not to individual alpha frequency
Source: PLoS One. 2017 Jun 12;12(6):e0178934. doi: 10.1371/journal.pone.0178934 (PMC5467839; doi:10.1371/journal.pone.0178934)
Supplement: S2 File — Listed are the settings for optional baseline parameters as used for data analysis in the present study. (DOCX) [file pone.0178934.s003.docx]

**S2 File. Settings for optional baseline parameters used in EEGLAB function newtimef.** Listed are the settings for optional baseline parameters as used for data analysis in the present study.

`baseline’ = [-200 0]

`powbase’ = default 🡪 from data

`basenorm’ = default 🡪 divide by the average power across trials at each frequency

`trialbase’ = default 🡪 perform baseline division in the trial average
