# Supplementary material for: Individual differences in dual-target RSVP task performance relate to entrainment but not to individual alpha frequency
Source: PLoS One. 2017 Jun 12;12(6):e0178934. doi: 10.1371/journal.pone.0178934 (PMC5467839; doi:10.1371/journal.pone.0178934)
Supplement: S1 Table — Correlation coefficients and p-values for 10 Hz ITC with T1 identification rate and T2|T1 lag 7 identification rate. (DOCX) [file pone.0178934.s004.docx]

|  | T1 | T2\|T1 lag 7 |
| --- | --- | --- |
| 10 Hz ITC | r = .33  p = .018 | r = .39  p = .005 |
| 10 Hz ITC outliers removed | r = .28  p =.047 | r = .35  p = .015 |
| 10 Hz ITC with  trial number partialed out | r = .31  p = .03 | r = .37  p = .008 |

**S1 Table.** **Correlation coefficients and *p*-values**. Correlation coefficients and *p*-values for 10 Hz ITC with T1 identification rate and T2|T1 lag 7 identification rate.
